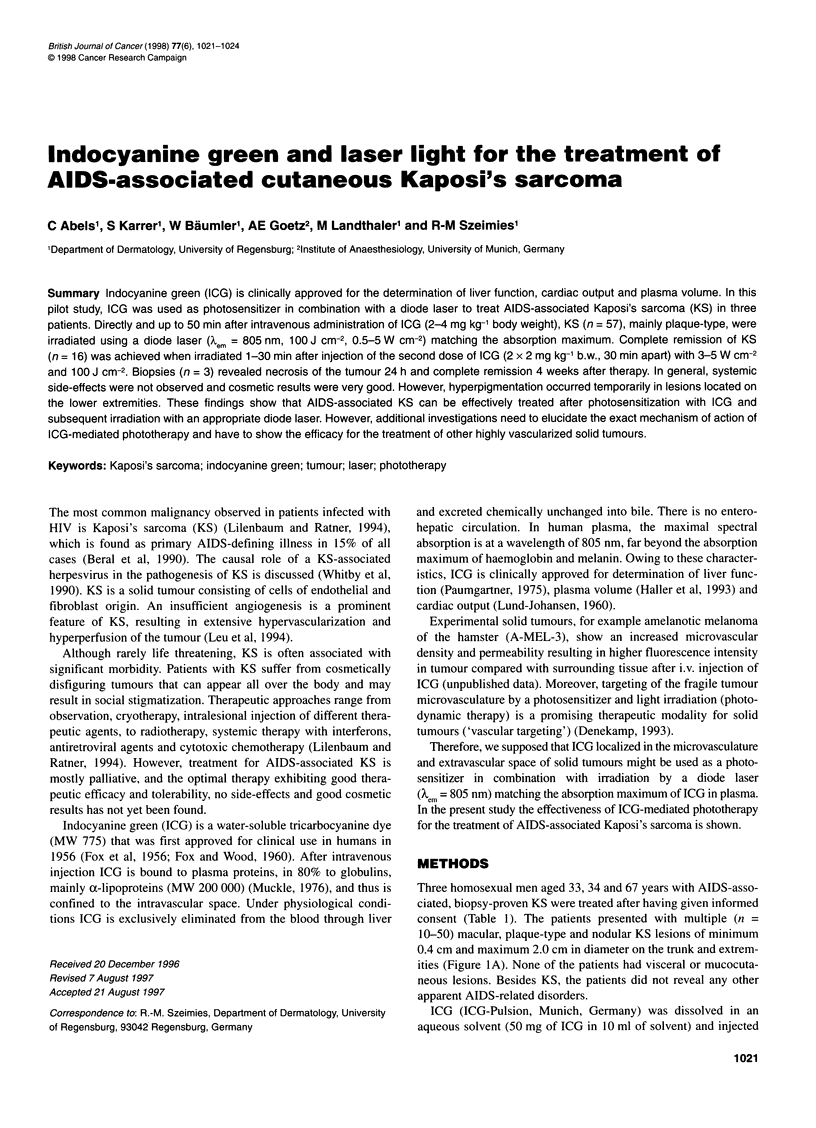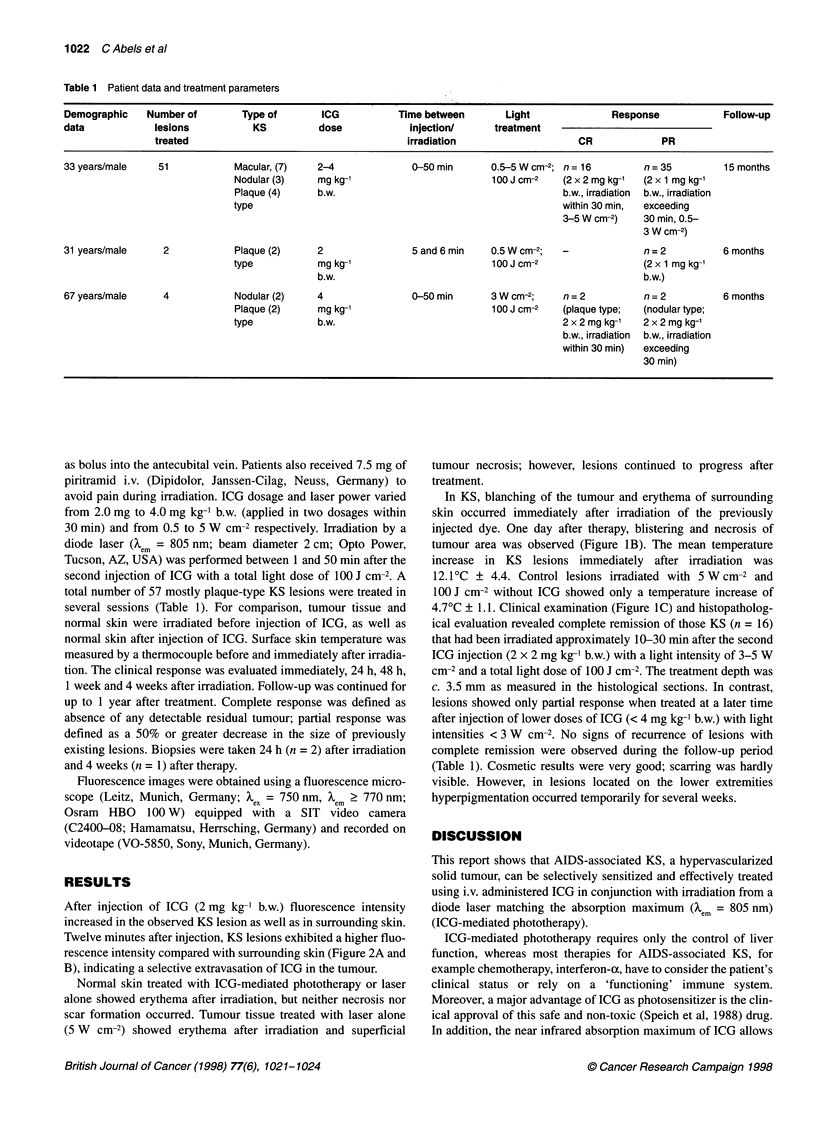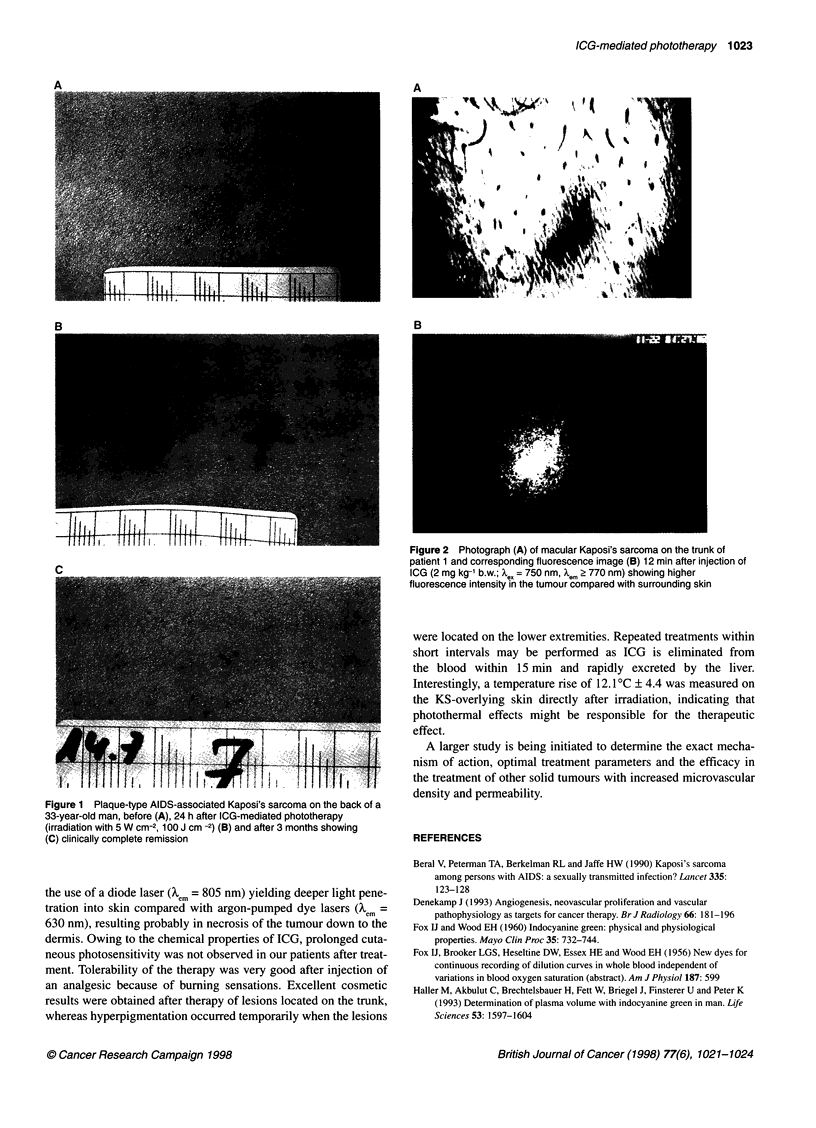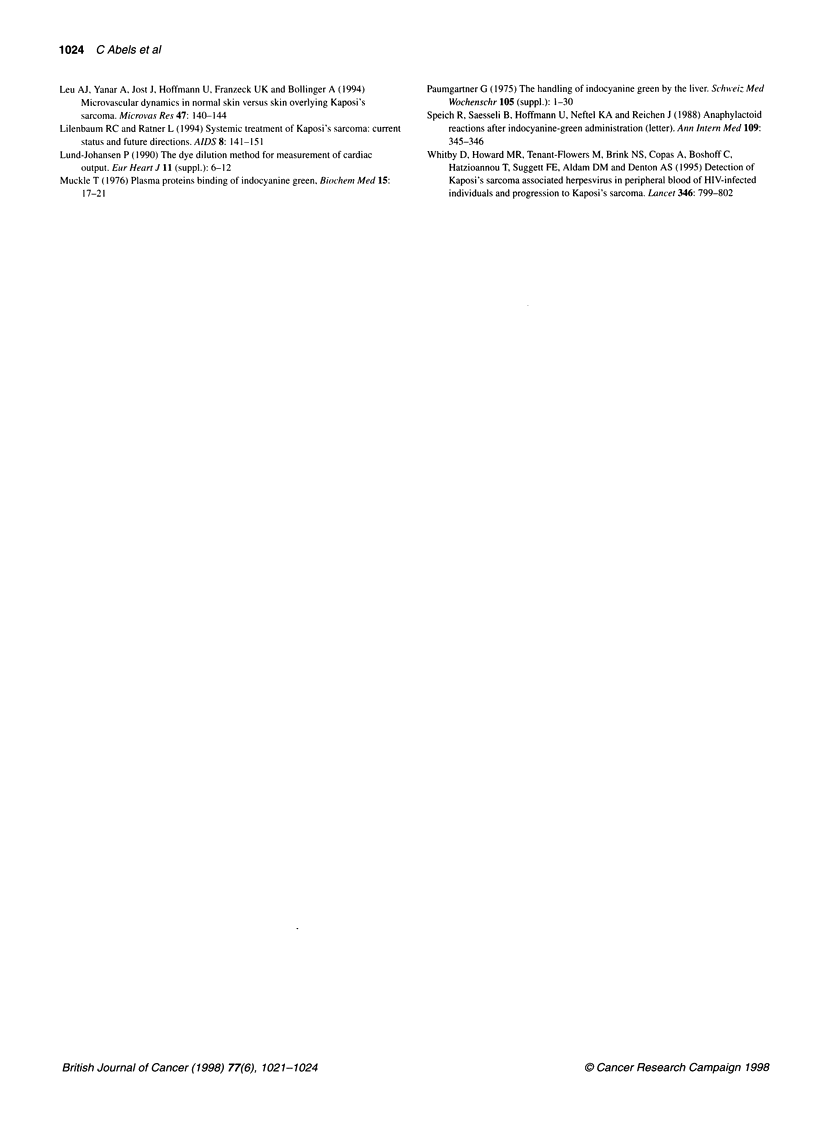# Indocyanine green and laser light for the treatment of AIDS-associated cutaneous Kaposi's sarcoma.

**DOI:** 10.1038/bjc.1998.168

**Published:** 1998-03

**Authors:** C. Abels, S. Karrer, W. BÃ¤umler, A. E. Goetz, M. Landthaler, R. M. Szeimies

**Affiliations:** Department of Dermatology, University of Regensburg, Germany.

## Abstract

**Images:**


*British Journal of Cancer* (1998) **77**(6), 1021–1024
© 1998 Cancer Research Campaign

### Indocyanine green and laser light for the treatment of AIDS-associated cutaneous Kaposi's sarcoma

**C Abels[1], S Karrer[1], W Bäumler[1], AE Goetz[2], M Landthaler[1] and R-M Szeimies[1]**

[1]Department of Dermatology, University of Regensburg; [2]Institute of Anaesthesiology, University of Munich, Germany

**Summary** Indocyanine green (ICG) is clinically approved for the determination of liver function, cardiac output and plasma volume. In this pilot study, ICG was used as photosensitizer in combination with a diode laser to treat AIDS-associated Kaposi's sarcoma (KS) in three patients. Directly and up to 50 min after intravenous administration of ICG (2–4 mg $kg^{-1}$ body weight), KS ($n$ = 57), mainly plaque-type, were irradiated using a diode laser ($\lambda_{em}$ = 805 nm, 100 J $cm^{-2}$, 0.5–5 W $cm^{-2}$) matching the absorption maximum. Complete remission of KS ($n$ = 16) was achieved when irradiated 1–30 min after injection of the second dose of ICG (2 × 2 mg $kg^{-1}$ b.w., 30 min apart) with 3–5 W $cm^{-2}$ and 100 J $cm^{-2}$. Biopsies ($n$ = 3) revealed necrosis of the tumour 24 h and complete remission 4 weeks after therapy. In general, systemic side-effects were not observed and cosmetic results were very good. However, hyperpigmentation occurred temporarily in lesions located on the lower extremities. These findings show that AIDS-associated KS can be effectively treated after photosensitization with ICG and subsequent irradiation with an appropriate diode laser. However, additional investigations need to elucidate the exact mechanism of action of ICG-mediated phototherapy and have to show the efficacy for the treatment of other highly vascularized solid tumours.

**Keywords:** Kaposi's sarcoma; indocyanine green; tumour; laser; phototherapy

The most common malignancy observed in patients infected with HIV is Kaposi's sarcoma (KS) (Lilenbaum and Ratner, 1994), which is found as primary AIDS-defining illness in 15% of all cases (Beral et al, 1990). The causal role of a KS-associated herpesvirus in the pathogenesis of KS is discussed (Whitby et al, 1990). KS is a solid tumour consisting of cells of endothelial and fibroblast origin. An insufficient angiogenesis is a prominent feature of KS, resulting in extensive hypervascularization and hyperperfusion of the tumour (Leu et al, 1994).

Although rarely life threatening, KS is often associated with significant morbidity. Patients with KS suffer from cosmetically disfiguring tumours that can appear all over the body and may result in social stigmatization. Therapeutic approaches range from observation, cryotherapy, intralesional injection of different therapeutic agents, to radiotherapy, systemic therapy with interferons, antiretroviral agents and cytotoxic chemotherapy (Lilenbaum and Ratner, 1994). However, treatment for AIDS-associated KS is mostly palliative, and the optimal therapy exhibiting good therapeutic efficacy and tolerability, no side-effects and good cosmetic results has not yet been found.

Indocyanine green (ICG) is a water-soluble tricarbocyanine dye (MW 775) that was first approved for clinical use in humans in 1956 (Fox et al, 1956; Fox and Wood, 1960). After intravenous injection ICG is bound to plasma proteins, in 80% to globulins, mainly α-lipoproteins (MW 200 000) (Muckle, 1976), and thus is confined to the intravascular space. Under physiological conditions ICG is exclusively eliminated from the blood through liver and excreted chemically unchanged into bile. There is no enterohepatic circulation. In human plasma, the maximal spectral absorption is at a wavelength of 805 nm, far beyond the absorption maximum of haemoglobin and melanin. Owing to these characteristics, ICG is clinically approved for determination of liver function (Paumgartner, 1975), plasma volume (Haller et al, 1993) and cardiac output (Lund-Johansen, 1960).

Experimental solid tumours, for example amelanotic melanoma of the hamster (A-MEL-3), show an increased microvascular density and permeability resulting in higher fluorescence intensity in tumour compared with surrounding tissue after i.v. injection of ICG (unpublished data). Moreover, targeting of the fragile tumour microvasculature by a photosensitizer and light irradiation (photodynamic therapy) is a promising therapeutic modality for solid tumours ('vascular targeting') (Denekamp, 1993).

Therefore, we supposed that ICG localized in the microvasculature and extravascular space of solid tumours might be used as a photosensitizer in combination with irradiation by a diode laser ($\lambda_{em}$ = 805 nm) matching the absorption maximum of ICG in plasma. In the present study the effectiveness of ICG-mediated phototherapy for the treatment of AIDS-associated Kaposi's sarcoma is shown.

### METHODS

Three homosexual men aged 33, 34 and 67 years with AIDS-associated, biopsy-proven KS were treated after having given informed consent (Table 1). The patients presented with multiple ($n$ = 10–50) macular, plaque-type and nodular KS lesions of minimum 0.4 cm and maximum 2.0 cm in diameter on the trunk and extremities (Figure 1A). None of the patients had visceral or mucocutaneous lesions. Besides KS, the patients did not reveal any other apparent AIDS-related disorders.

ICG (ICG-Pulsion, Munich, Germany) was dissolved in an aqueous solvent (50 mg of ICG in 10 ml of solvent) and injected

*Received 20 December 1996*
*Revised 7 August 1997*
*Accepted 21 August 1997*

*Correspondence to*: R.-M. Szeimies, Department of Dermatology, University of Regensburg, 93042 Regensburg, Germany

**Table 1** Patient data and treatment parameters

| Demographic data | Number of lesions treated | Type of KS | ICG dose | Time between injection/ irradiation | Light treatment | Response | | Follow-up |
|---|---|---|---|---|---|---|---|---|
| | | | | | | CR | PR | |
| 33 years/male | 51 | Macular, (7) Nodular (3) Plaque (4) type | 2–4 mg kg$^{-1}$ b.w. | 0–50 min | 0.5–5 W cm$^{-2}$; 100 J cm$^{-2}$ | $n$ = 16 (2 × 2 mg kg$^{-1}$ b.w., irradiation within 30 min, 3–5 W cm$^{-2}$) | $n$ = 35 (2 × 1 mg kg$^{-1}$ b.w., irradiation exceeding 30 min, 0.5–3 W cm$^{-2}$) | 15 months |
| 31 years/male | 2 | Plaque (2) type | 2 mg kg$^{-1}$ b.w. | 5 and 6 min | 0.5 W cm$^{-2}$; 100 J cm$^{-2}$ | – | $n$ = 2 (2 × 1 mg kg$^{-1}$ b.w.) | 6 months |
| 67 years/male | 4 | Nodular (2) Plaque (2) type | 4 mg kg$^{-1}$ b.w. | 0–50 min | 3 W cm$^{-2}$; 100 J cm$^{-2}$ | $n$ = 2 (plaque type; 2 × 2 mg kg$^{-1}$ b.w., irradiation within 30 min) | $n$ = 2 (nodular type; 2 × 2 mg kg$^{-1}$ b.w., irradiation exceeding 30 min) | 6 months |

as bolus into the antecubital vein. Patients also received 7.5 mg of piritramid i.v. (Dipidolor, Janssen-Cilag, Neuss, Germany) to avoid pain during irradiation. ICG dosage and laser power varied from 2.0 mg to 4.0 mg kg$^{-1}$ b.w. (applied in two dosages within 30 min) and from 0.5 to 5 W cm$^{-2}$ respectively. Irradiation by a diode laser ($\lambda_{em}$ = 805 nm; beam diameter 2 cm; Opto Power, Tucson, AZ, USA) was performed between 1 and 50 min after the second injection of ICG with a total light dose of 100 J cm$^{-2}$. A total number of 57 mostly plaque-type KS lesions were treated in several sessions (Table 1). For comparison, tumour tissue and normal skin were irradiated before injection of ICG, as well as normal skin after injection of ICG. Surface skin temperature was measured by a thermocouple before and immediately after irradiation. The clinical response was evaluated immediately, 24 h, 48 h, 1 week and 4 weeks after irradiation. Follow-up was continued for up to 1 year after treatment. Complete response was defined as absence of any detectable residual tumour; partial response was defined as a 50% or greater decrease in the size of previously existing lesions. Biopsies were taken 24 h ($n$ = 2) after irradiation and 4 weeks ($n$ = 1) after therapy.

Fluorescence images were obtained using a fluorescence microscope (Leitz, Munich, Germany; $\lambda_{ex}$ = 750 nm, $\lambda_{em}$ ≥ 770 nm; Osram HBO 100 W) equipped with a SIT video camera (C2400–08; Hamamatsu, Herrsching, Germany) and recorded on videotape (VO-5850, Sony, Munich, Germany).

### RESULTS

After injection of ICG (2 mg kg$^{-1}$ b.w.) fluorescence intensity increased in the observed KS lesion as well as in surrounding skin. Twelve minutes after injection, KS lesions exhibited a higher fluorescence intensity compared with surrounding skin (Figure 2A and B), indicating a selective extravasation of ICG in the tumour.

Normal skin treated with ICG-mediated phototherapy or laser alone showed erythema after irradiation, but neither necrosis nor scar formation occurred. Tumour tissue treated with laser alone (5 W cm$^{-2}$) showed erythema after irradiation and superficial tumour necrosis; however, lesions continued to progress after treatment.

In KS, blanching of the tumour and erythema of surrounding skin occurred immediately after irradiation of the previously injected dye. One day after therapy, blistering and necrosis of tumour area was observed (Figure 1B). The mean temperature increase in KS lesions immediately after irradiation was 12.1°C ± 4.4. Control lesions irradiated with 5 W cm$^{-2}$ and 100 J cm$^{-2}$ without ICG showed only a temperature increase of 4.7°C ± 1.1. Clinical examination (Figure 1C) and histopathological evaluation revealed complete remission of those KS ($n$ = 16) that had been irradiated approximately 10–30 min after the second ICG injection (2 × 2 mg kg$^{-1}$ b.w.) with a light intensity of 3–5 W cm$^{-2}$ and a total light dose of 100 J cm$^{-2}$. The treatment depth was *c.* 3.5 mm as measured in the histological sections. In contrast, lesions showed only partial response when treated at a later time after injection of lower doses of ICG (< 4 mg kg$^{-1}$ b.w.) with light intensities < 3 W cm$^{-2}$. No signs of recurrence of lesions with complete remission were observed during the follow-up period (Table 1). Cosmetic results were very good; scarring was hardly visible. However, in lesions located on the lower extremities hyperpigmentation occurred temporarily for several weeks.

### DISCUSSION

This report shows that AIDS-associated KS, a hypervascularized solid tumour, can be selectively sensitized and effectively treated using i.v. administered ICG in conjunction with irradiation from a diode laser matching the absorption maximum ($\lambda_{em}$ = 805 nm) (ICG-mediated phototherapy).

ICG-mediated phototherapy requires only the control of liver function, whereas most therapies for AIDS-associated KS, for example chemotherapy, interferon-α, have to consider the patient's clinical status or rely on a 'functioning' immune system. Moreover, a major advantage of ICG as photosensitizer is the clinical approval of this safe and non-toxic (Speich et al, 1988) drug. In addition, the near infrared absorption maximum of ICG allows

© Cancer Research Campaign 1998

**Figure 1** Plaque-type AIDS-associated Kaposi's sarcoma on the back of a 33-year-old man, before (**A**), 24 h after ICG-mediated phototherapy (irradiation with 5 W cm$^{-2}$, 100 J cm $^{-2}$) (**B**) and after 3 months showing (**C**) clinically complete remission the use of a diode laser ($\lambda_{em}$ = 805 nm) yielding deeper light penetration into skin compared with argon-pumped dye lasers ($\lambda_{em}$ = 630 nm), resulting probably in necrosis of the tumour down to the dermis. Owing to the chemical properties of ICG, prolonged cutaneous photosensitivity was not observed in our patients after treatment. Tolerability of the therapy was very good after injection of an analgesic because of burning sensations. Excellent cosmetic results were obtained after therapy of lesions located on the trunk, whereas hyperpigmentation occurred temporarily when the lesions

**Figure 2** Photograph (**A**) of macular Kaposi's sarcoma on the trunk of patient 1 and corresponding fluorescence image (**B**) 12 min after injection of ICG (2 mg kg$^{-1}$ b.w.; $\lambda_{ex}$ = 750 nm, $\lambda_{em}$ ≥ 770 nm) showing higher fluorescence intensity in the tumour compared with surrounding skin were located on the lower extremities. Repeated treatments within short intervals may be performed as ICG is eliminated from the blood within 15 min and rapidly excreted by the liver. Interestingly, a temperature rise of 12.1°C ± 4.4 was measured on the KS-overlying skin directly after irradiation, indicating that photothermal effects might be responsible for the therapeutic effect.

A larger study is being initiated to determine the exact mechanism of action, optimal treatment parameters and the efficacy in the treatment of other solid tumours with increased microvascular density and permeability.

### REFERENCES

Beral V, Peterman TA, Berkelman RL and Jaffe HW (1990) Kaposi's sarcoma among persons with AIDS: a sexually transmitted infection? *Lancet* **335**: 123–128

Denekamp J (1993) Angiogenesis, neovascular proliferation and vascular pathophysiology as targets for cancer therapy. *Br J Radiology* **66**: 181–196

Fox IJ and Wood EH (1960) Indocyanine green: physical and physiological properties. *Mayo Clin Proc* **35**: 732–744.

Fox IJ, Brooker LGS, Heseltine DW, Essex HE and Wood EH (1956) New dyes for continuous recording of dilution curves in whole blood independent of variations in blood oxygen saturation (abstract). *Am J Physiol* **187**: 599

Haller M, Akbulut C, Brechtelsbauer H, Fett W, Briegel J, Finsterer U and Peter K (1993) Determination of plasma volume with indocyanine green in man. *Life Sciences* **53**: 1597–1604

© Cancer Research Campaign 1998

Leu AJ, Yanar A, Jost J, Hoffmann U, Franzeck UK and Bollinger A (1994) Microvascular dynamics in normal skin versus skin overlying Kaposi's sarcoma. *Microvas Res* **47**: 140–144

Lilenbaum RC and Ratner L (1994) Systemic treatment of Kaposi's sarcoma: current status and future directions. *AIDS* **8**: 141–151

Lund-Johansen P (1990) The dye dilution method for measurement of cardiac output. *Eur Heart J* **11** (suppl.): 6–12

Muckle T (1976) Plasma proteins binding of indocyanine green, *Biochem Med* **15**: 17–21

Paumgartner G (1975) The handling of indocyanine green by the liver. *Schweiz Med Wochenschr* **105** (suppl.): 1–30

Speich R, Saesseli B, Hoffmann U, Neftel KA and Reichen J (1988) Anaphylactoid reactions after indocyanine-green administration (letter). *Ann Intern Med* **109**: 345–346

Whitby D, Howard MR, Tenant-Flowers M, Brink NS, Copas A, Boshoff C, Hatzioannou T, Suggett FE, Aldam DM and Denton AS (1995) Detection of Kaposi's sarcoma associated herpesvirus in peripheral blood of HIV-infected individuals and progression to Kaposi's sarcoma. *Lancet* **346**: 799–802

© Cancer Research Campaign 1998